# Removal of Mercury by Foam Fractionation Using Surfactin, a Biosurfactant

**DOI:** 10.3390/ijms12118245

**Published:** 2011-11-21

**Authors:** Hau-Ren Chen, Chien-Cheng Chen, A. Satyanarayana Reddy, Chien-Yen Chen, Wun Rong Li, Min-Jen Tseng, Hung-Tsan Liu, Wei Pan, Jyoti Prakash Maity, Shashi B. Atla

**Affiliations:** 1 Department of Life Science, National Chung Cheng University, 168 University Road, Minhsiung, Chiayi 621, Taiwan; E-Mails: biohrc@ccu.edu.tw (H.-R.C.); biomjt@ccu.edu.tw (M.-J.T.); 2 Department of Biotechnology, National Kaohsiung Normal University, No. 62, Shenjhong Road, Yanchao Township, Kaohsiung County 82444, Taiwan; E-Mail: xavierch2000@yahoo.com; 3 Department of Earth and Environmental Sciences, National Chung Cheng University, 168 University Road, Minhsiung, Chiayi 621, Taiwan; E-Mails: akurireddy@gmail.com (A.S.R.); gs_tsan@yahoo.com.tw (H.-T.L.); jyoti_maity@yahoo.com (J.P.M.); shashi_org@yahoo.com (S.B.A.); 4 Department of Engineering, University of Cambridge, Trumpington Street, Cambridge CB2 1PZ, UK; 5 Departments of Urology, Mackay Memorial Hospital, 92, Section 2 Chung San North Road, Taipei 10449, Taiwan; E-Mail: jessica@ms1.mmh.org.tw; 6 Department of Physics, National Chung Cheng University, 168 University Road, Minhsiung, Chiayi 621, Taiwan; E-Mail: wpan@ccu.edu.tw

**Keywords:** mercury removal, foam fractionation, biosurfactant, Surfactin

## Abstract

The separation of mercury ions from artificially contaminated water by the foam fractionation process using a biosurfactant (surfactin) and chemical surfactants (SDS and Tween-80) was investigated in this study. Parameters such as surfactant and mercury concentration, pH, foam volume, and digestion time were varied and their effects on the efficiency of mercury removal were investigated. The recovery efficiency of mercury ions was highly sensitive to the concentration of the surfactant. The highest mercury ion recovery by surfactin was obtained using a surfactin concentration of 10 × CMC, while recovery using SDS required < 10 × CMC and Tween-80 >10 × CMC. However, the enrichment of mercury ions in the foam was superior with surfactin, the mercury enrichment value corresponding to the highest metal recovery (10.4%) by surfactin being 1.53. Dilute solutions (2-mg L^−1^ Hg^2+^) resulted in better separation (36.4%), while concentrated solutions (100 mg L^−1^) enabled only a 2.3% recovery using surfactin. An increase in the digestion time of the metal solution with surfactin yielded better separation as compared with a freshly-prepared solution, and an increase in the airflow rate increased bubble production, resulting in higher metal recovery but low enrichment. Basic solutions yielded higher mercury separation as compared with acidic solutions due to the precipitation of surfactin under acidic conditions.

## 1. Introduction

Mercury is found in several forms, including metal and salts [1]. Most of the mercury in the environment is present as elemental mercury, which tends to stay airborne. Volcanic degassing is thought to be the largest source of ocean and atmospheric mercury. Alkali and metal processing, incineration of coal, medical and other waste, and mining of gold and mercury contribute greatly to the mercury concentration in some areas, but atmospheric deposition is the dominant source of mercury. Mercury tends to be more reactive to form methylmercury [2], which is mainly produced by microorganisms present in water and soil. Mercury pollution has caused a sharp rise in mercury levels in many fish species, increasing the danger to humans that consume them. Mercury, which can travel thousands of miles from its original source, damages the central nervous system, and is especially dangerous to pregnant women and babies [3]. Mercury compounds differ greatly in their toxicity and environmental mobility [4]. Being lipid-soluble, certain mercury salts, such as HgCl_2_, diffuse through biological cell membranes, where methyl mercury may form (Jay *et al*., 2000; Lee and Jiang, 2000) [5,6]. Once methylated, mercury binds more easily to proteins and accumulates in living organisms.

A number of methods for the removal of mercury contaminating civil and industrial waste have been reported in the literature. Adsorption of mercury by activated carbons and other solid materials is commonly adopted [7–9]. The other methods includes chemical precipitation, ion exchange, cementation [10], coagulation and flocculation, complexation, biosorption [11,12], and membrane processes [13]. In addition, soil excavation and transport of contaminated soil to hazardous waste sites for landfilling, thermal extraction for volatile metals, electrokinetics, solidification/stabilization, vitrification, chemical oxidation, soil flushing, and bioremediation are other potential remediation methods for contaminated soil [14–16]. Membrane processes are reported but operate under a very high transmembrane pressure; micellar enhanced ultrafiltration (MEUF) processes have been adopted to overcome this problem [17,18]. Extraction of heavy metal ions from effluent water microemulsified systems has proved a good alternative due to its advantages over conventional solvent extraction [19]. Attempts are being made to decontaminate polluted soil and water, including techniques for on-site and off-site removal of contaminated soil. None of these methods is ideal for remediating contaminated soils, and often, more than one method may be necessary to optimize the cleanup effort. However, these methods are expensive and time-consuming, and hence alternative cost-effective, benign processes and use of renewable chemicals in remedial techniques need to be developed. Foam fractionation, is a cost-effective and simple separation process ideal for the removal of heavy metals from contaminated sites. This process is versatile and works better than the other techniques described, especially when the heavy metal concentration is low [20–22]. Foam fractionation methods for removal of heavy metals were also reported elsewhere [15,23,24]. This technique operates based on the fact that surface-active molecules tend to accumulate at the gas–liquid interface, and by bubbling air through the solution, the surface-active material is absorbed at the surface of the bubble and then separated from the solution. The metal constituent of the solution to be removed, if not already surface-active, can be made so through union with or adherence to a surface-active material by the formation of chelates, electrostatic interaction, or other mechanisms. The foam fractionation method is regarded as an effective tool for controlling mercury in the environment and has been adopted by the United States Environmental Protection Agency [20,21,24] It was also reported that the presence of proper chelating agents enhances the separation of mercury ions by foam fractionation [22].

Remediation of heavy metals from contaminated soil/industrial waste streams necessitates the use of surfactants, which by complexation with metal ions mobilize or increase the availability of contaminants [25,26]. Although the use of bacteria or bacterial exopolymers for the complexation of metals from waste streams has been extensively studied and reviewed [27,28] the use of smaller biomolecules for metal complexation is of special interest for application in heavy metal remediation [29]. Use of various biosurfactants for the removal of heavy metal contaminants in soil and water treatment processes were reported [15,30–36]. Biosurfactants have similar emulsification properties to chemical surfactants and are nontoxic; they are also biodegradable and can be biodegraded, and they do not remain in the environment and are benign to the environment. Different types of biosurfactants have been used for the removal of heavy metals from sediment [32]. Biosurfactants have the following advantages over chemical surfactants: lower toxicity, higher biodegradability, better environmental compatibility, higher foaming property, higher selectivity for metal ions and organic compounds, more tolerant to pH, salt and temperature variation, the ability to be synthesized from renewable sources, and in some cases, less expensive. Biosurfactants can be classified as glycolipids, lipopeptides, phospholipids, fatty acids, and neutral lipids [32]. Surfactin, a biosurfactant, is a bacterial cyclic lipopeptide renowned for its exceptional surfactant power, as it lowers the surface tension of water from 72 to 27 mN/m at concentrations as low as 20 μM [37].

The removal of mercury from artificially contaminated water using surfactin was investigated in this study. Foam fractionation was employed for this purpose, due to the importance of this process in controlling the level of mercury in the environment, as reported by the United States environmental protection agency guidelines [21,22]. We focused on the use of surfactin in the recovery of mercury in a batch operation, and the effects of surfactin concentration, mercury concentration, foaming time, digestion time, airflow, and pH of the solution on the enrichment and recovery of mercury were investigated. The performance of surfactin in the heavy metal removal process was also compared with those of chemical counterparts, such as sodium dodecyl sulfate (SDS) and Tween-80 (polyoxyethylene sorbitan monooleate). Foam drainage characteristics of surfactant transport between the rising and falling streams during fractionation was explained with help of models and he validity of the models has been demonstrated experimentally for a foam fractionation system continuously separating CPC from a solution of constant concentration under total reflux [38].

## 2. Results and Discussion

Surfactin, a biosurfactant, and chemical surfactants, SDS and Tween-80, were used in this study in order to demonstrate the superiority of the biosurfactant over chemical counterparts in the removal of mercury from contaminated water. The binding/complexation capacity of the surfactants with mercury ions, separating them depends on various parameters. Surfactin and mercury concentration, pH of the solution, foaming time, and digestion time determined the metal removal efficiency.

### 2.1. Effect of Surfactant Concentration

**Figure 1:**
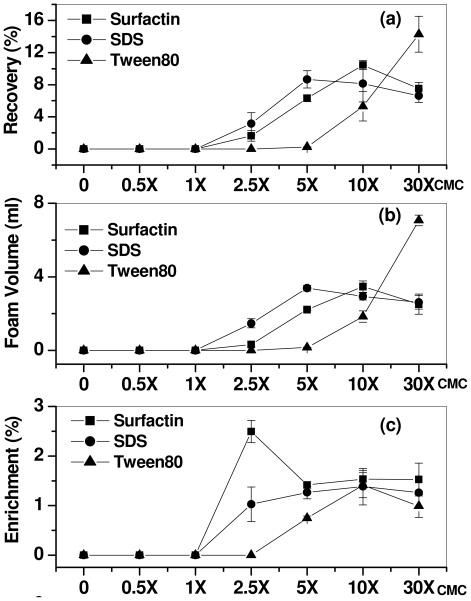
Effect of surfactants concentration on mercury removal by the in foam fractionation in terms of (**a**) percentage recovery (**b**) foam volume (**c**) enrichment.

**Figure 2:**
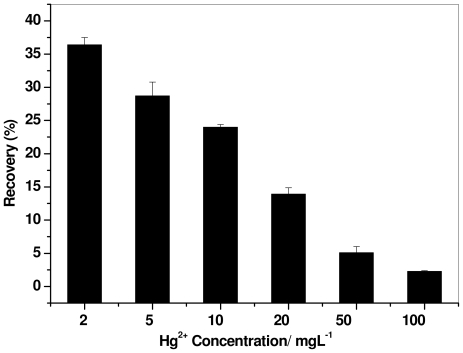
Effect of mercury concentration on the recovery efficiency using surfactin (10 × critical micelle concentration (CMC)) solution at a flow rate of 1 L min^−1^ and foaming time of 3 min.

Figure 1 shows the mercury recovery by various surfactants as a function of their concentration. The enrichment, foam volume and recovery percentage of mercury were investigated. The concentration of mercury in the solution was 10 mg L^−1^, and the foam was collected after 3 min of initiation of airflow (1 L min^−1^). The concentration of the surfactants varied by orders of 0.5, 1, 2, 5, 5, 10, and 30 times the critical micelle concentration (CMC) of the surfactants investigated. The CMC values of the surfactants used in this study are 7.5 × 10^−3^, 8.2 and 1.2 × 10^−2^ mM for surfactin, SDS, and Tween-80, respectively [39–41]. The recovery of mercury was found to be sensitive to the surfactant concentration. No foam was accumulated in the stipulated time of 3 min in the collection flask when 0.5 and 1 × CMC surfactin and SDS were used; this could be due to insufficient volume of the foam generated in the solution, and the small amount of foam generated did not travel through the J-tube to reach the collector flask. Similarly, in case of Tween-80, the foam did not reach the collector flask, even with surfactant with 2.5 × CMC. Figure 2(a) shows that the highest mercury recovery was obtained by surfactin at 10 × CMC, while SDS and Tween-80 recovered highest mercury recovery at <10× and >10 × CMC, respectively. Recovery of the mercury by the surfactants at given concentrations was increased with the increase of foam volume (Figure 2(b)). However, the enrichment of mercury ions in the foam was superior with surfactin compared with chemical surfactants and was highest at lower concentrations (2.5 × CMC). The anionic surfactants (surfactin and SDS) recovered higher mercury at low concentrations compared to Tween-80; this may due to the electrostatic interaction between negatively charged functional groups (carboxylates in surfactin and sulfate in SDS) and mercury ions (Hg^2+^) resulting in the formation of a complex in the form of micelle [42]. The maximum recovery of mercury ions (10.4%) using surfactin was observed at 10 × CMC, whereas that of SDS at the same concentration was 8.14%; the highest mercury recovery for SDS (8.8%) was obtained at 5 × CMC. The highest recovery rate overall, *i.e.*, 14.2%, was observed with Tween-80, but this required a concentration of 30 × CMC. The mercury enrichment value corresponding to the highest metal recovery by surfactin (10.4%) was 1.53; this may be due to the presence of two carboxylate functional groups of surfactin, which can bind to the metal ions, whereas only one sulfate group is present in the SDS molecule. It is also known that, as the concentration of surfactant increases in multiples of the CMC, vesicle structures outnumber the micelles; in addition the structure and aggregate numbers also change. The difference in the recovery and enrichment values at the given surfactant concentration may be due to variable liquid concentration in the foam that varies significantly with the nature of the surfactants used. Hence, a higher concentration of Tween-80 was required for mercury recovery compared anionic surfactants. These results indicated that the complexation efficiency and separation of surfactin along with metal ions strongly depend on the nature and concentration of the surfactant. Surfactin was found to form micelles at very low concentrations, but resulted in a high mercury removal (10.4% at 10 × CMC) [39,40]. The following experiments were also performed using surfactin at a concentration of 10 × CMC, in which various parameters were altered in order to investigate the factors affecting the recovery of mercury from the solution.

### 2.2. Effect of Mercury Concentration

Figure 2 presents the effect of the mercury concentration on its recovery using surfactin at a flow rate of 1 L min^−1^ and a foaming time of 3 min. Recovery was performed at mercury concentrations of 2, 5, 10, 20, 50, and 100 mg L^−1^ and in a 10 × CMC surfactin solution. The metal recovery was found to decrease with increasing mercury concentration. The highest recovery observed was 36.4% from 2 mg L^−1^ mercury solution and 2.29% from100 mg L^−1^ solution. It has already been reported that the percentage recovery of mercury by foam fractionation was better at low concentrations [24]. The effective complexation ratio of surfactin to metal decreased with the increase of mercury concentration in the solution. Theoretically, in the absence of interfering ions, the molar ratio of surfactin to Hg^2+^ is expected to be 0.5 due to complexation of two Hg^2+^ ions with one surfactin molecule (each surfactin molecule contains two carboxylate groups); however, the surfactin to Hg^2+^ ratio in 2 mg L^−1^ solution was found to be 3.75 that decreased to 0.075 for a 100 mg L^−1^ solution. This is probably limited by the micelle formation in surfactin solution (10 × CMC), which was able to form a complex effectively from lower concentration of mercury ions carry to the foam collector.

### 2.3. Foaming Time Course

**Figure 3:**
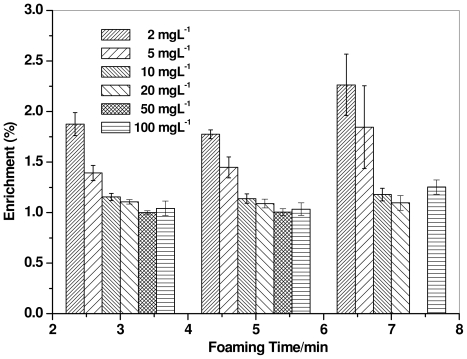
Effect of foaming time on the mercury enrichment by a surfactin (10 × CMC) solution at a flow rate of 1 L min^−1^.

It is also known that, in the foam fractionation method, foam volume affects the metal ion enrichment by carrying a more number of surfactant–metal complex entities. Hence, foam was collected at intervals of two minutes using different concentrations (2, 5, 10, 20, 50, and 100 mg L^−1^) of mercury ions in the solution in order to investigate the effect of foaming time on mercury recovery. The concentration of surfactin and the airflow were fixed at 10 × CMC and 1 L min^−1^, respectively. Figure 3 shows that the foam collected after 6 min enriched with mercury by 2.26% from the surfactin solution containing 2 mg L^−1^ Hg^2+^. It is presumed that the low enrichment after 3 (1.87%) and 5 (1.78%) min of foam collection was due to the large number of foam bubbles collected in the flask. Thus, foam volume is also an important factor affecting mercury ion enrichment in the foam fractionation method. When the mercury ion concentration was 2 and 5 mg L^−1^, the enrichment was higher compared to other concentrations, and thereafter remained nearly constant with increased mercury ion concentration up to 100 mg L^−1^ [43].

### 2.4. Effect of Digestion Time

**Figure 4:**
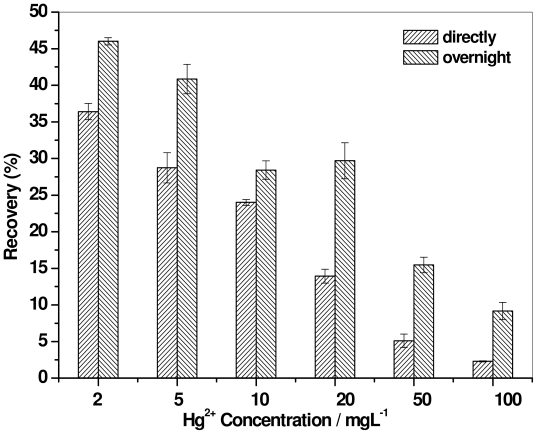
Comparison of digestion time of mercury in surfactin solution (10 × CMC) on recovery with different concentrations of mercury.

The effect of the digestion time, which in turn influences the complexation of the metal ions with the surfactant, is also important in the recovery of heavy metals. The effect of digestion time of mercury in surfactin solution was investigated for mercury removal by conducting experiment with fresh and overnight solutions. Figure 4 presents enrichment of mercury from the surfactin solution (10 × CMC) of different mercury concentrations and foaming time of 7 min. Apparently, there was considerable recovery of mercury ions from the solution digested overnight; the highest recovery was 46%, when the mercury ion concentration was 2 mg L^−1^, and recovery was higher by a magnitude of four than that observed at the highest mercury ion concentration, *i.e.*, 100 mg L^−1^. Thus, the removal efficiency of mercury ions increased when mercury ions were digested overnight in the surfactin solution, due to the stable complex formation of mercury ions with surfactin. It was conjectured that, in order for the highest recovery % to be obtained, each functional group must bind a mercury ion. It has previously been reported that a prolonged digestion time of approximately 36 hours for contaminated soil with rhamnolipid solution yielded recovery of heavy metals up to 92% [31].

### 2.5. Effect of Airflow Rate

**Figure 5:**
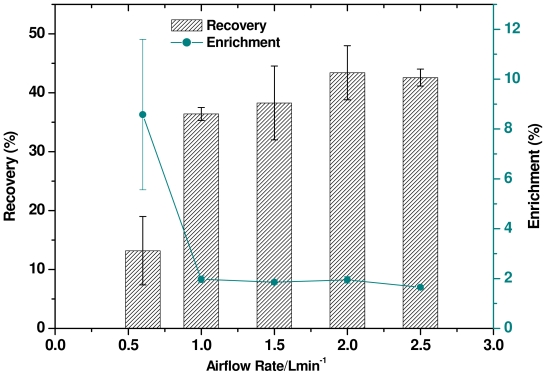
Effect of airflow rate on mercury recovery and enrichment using surfactin solution (10 × CMC) and 2 mg L^−1^ mercury.

Figure 5 shows the results of varying the airflow rate to generate foam of differing bubble size and stability. The effect of different flow rates using 10 × CMC surfactin and 2 mg L^−1^ mercury was studied in order to assess the influence of airflow on the recovery of mercury ions. The airflow rate was varied from 0.6 to 2.5 L min^−1^. The foaming time and the time required to pass through the J-type column was slow when the airflow rate was 0.6 L min^−1^, and a large number of bubbles in the foam were broken or remained in the J-type column and didn’t reach the foam collector; hence, this airflow rate resulted in a lower volume of foam in the collector, the analysis of which could be erroneous. Mercury ion enrichment at this low airflow rate was highest at 8.6%, but the mercury ion recovery was only 13.17%. The efficiency of mercury ion enrichment was almost constant when the airflow rate was above 1.0 L min^−1^, while mercury ion recovery was 43.4% and 42.6% when the airflow rate was 2.0 and 2.5 L min^−1^, respectively. Qua *et al.* reported that an increase in airflow rate with SDS decreased the enrichment ratio of Cd^2+^, while the % removal of the metal ions increased [44]. These results can be explained based on the fact that with an increase in airflow rate, a greater amount of the liquid could be transported into the foam and adsorbed onto the bubble surfaces, thereby increasing bubble production, resulting in higher recovery but low enrichment.

### 2.6. Effect of pH

**Figure 6:**
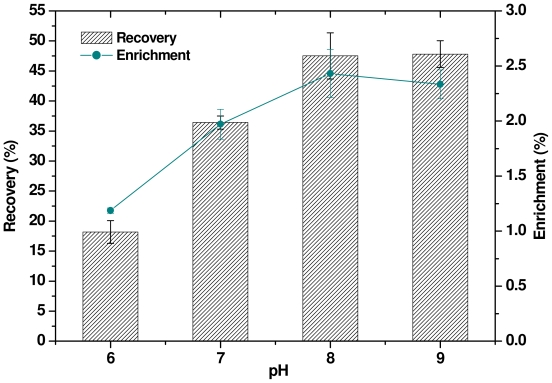
Effect of pH on mercury recovery and enrichment using surfactin solution (10 × CMC) and 2 mg L^−1^ mercury.

It is known that the pH of the surfactin solution influences the enrichment and recovery of heavy metal ions due to various factors. The effect of pH on the removal of mercury ions is shown in Figure 6, from which it can be seen that the mercury ion recovery was 47.5% and 47.8% at pH values of 8 and 9, respectively, and the mercury ion enrichment was about 2.43 and 2.33 at those pH values. However, mercury ion recovery was only 18.2% when the pH of the surfactin solution was decreased to 6, with an enrichment of 1.2. In addition, a lower pH of the surfactin solution may result in precipitation, thereby limiting the efficiency of the mercury removal process. It has also been reported that chelates of Hg^2+^ such as Cl^−^ and OH^−^ chelates are quite stable, leading to poor or no separation using sodium lauryl sulfate when the pH is reduced by HNO_3_ or kept alkaline [24]. Hence, the low recovery and enrichment values of mercury under acidic conditions could be attributed to undissociated carboxyl groups to form complexation with surfactin molecules. However, surfactin resulted in better separation of metal ions with increased pH. These results show that the efficiency of separation is closely related to pH, as well as to the concentration of the positively-charged mercury-containing species.

## 3. Experimental Section

### 3.1. Surfactin Production

In this study, surfactin was produced by wild-type *Bacillus subtilis* (BBK006) in culture media containing glucose as the sole carbon source. Production of surfactin using B. subtilis has been reported elsewhere [45,46]. Typically, a loop of bacteria grown on an LB agar plate was added to M9 medium (with 0.2% glucose) as a seed culture to make 30 mL of solution in a 50-mL tube. This was incubated for 24 h on an orbital shaker (200 rpm) at 37 °C in order to produce a seed culture. The exact composition of M9 medium was reported elsewhere [47]. Prior to sterilization, the medium pH was adjusted to 7.0 with 0.5 M NaOH. The medium was then sterilized at 121 °C for 20 min without glucose, which was filtersterilized (Millipore membrane PVDF, 0.22 μm filter unit; Millipore, Watford, UK). 300 mL M9 medium in a 500-mL Erlenmeyer flask was mixed with 0.2% glucose to produce surfactin. After 24 h of fermentation, the biomass was centrifuged (12,000 rpm, 10 min), followed by addition of 1 M HCl to adjust the pH of the supernatant solution to below 2.0. The precipitate was collected after centrifugation (12,000 g, 10 min) and the surfactin was purified as previously reported [40] by extraction with dichloromethane, dissolving in milli Q water to the required concentration, and stored at 4 °C. SDS and Tween-80 solutions were prepared at the time of experiment. The chemicals used in the growth media were purchased from Riedel-de Haën, GR, USA; the other surfactants, sodium dodecyl sulfate (98.5%) and Tween-80, were purchased from Sigma-Aldrich, Saint Louis, MO, USA.

### 3.2. Experimental Set Up

All experiments were conducted at room temperature (25 °C). The foam fractionation system used for removal of mercury consists of a glass J-type column (L = 400 and 100 mm, ID = 22 mm) connected to 1-L Erlenmeyer flasks at both the ends with rubber corks. Required concentrations (0.5, 1, 2.5, 5, 10 and 30 times of CMC of different surfactants (SDS, Tween-80 and surfactin) solution were taken in one of the Erlenmeyer flasks (connected to long arm) and connected to air pump) and foam was collected in another flask. The airflow rate (0.5, 1, 1.5, 2 and 2.5 L min^−1^) was controlled by a flow meter and the foam was collected in another flask to enable measurement of the concentration of mercury. The concentration of mercury in the artificially polluted water was varied from 2 to 100 mg L^−1^. The foam was generated from fresh and overnight digestion solutions. The time of foam collection was varied from 3 to 7 min. Final fate of the mercury was in the form of solution after bubbles collapsed in the collector flask.

### 3.3. Mercury Concentration Analysis

The mercury ion concentrations in the foam were analyzed using an atomic absorption spectrophotometer (AAS, Perkin Elmer Analyst 200, Shelton, CT, USA). The samples were digested in 3% HNO_3_ prior to the analysis. Mercury solution was diluted to 2 to 20 μmL^−1^ and the instrument was calibrated in this concentration range. All experiments were performed in triplicate, and the average of the results is presented. The metal removal efficiency was calculated by using the following formula, as reported elsewhere [48]:

(1) $$\text{Enrichment}=\frac{{\text{C}}_{\text{F}}\;(\text{ppm})}{{\text{C}}_{\text{R}}\;(\text{ppm})}$$

(2) $$\text{Recovery}\;(\%)=\frac{\text{CF}\;(\text{ppm})\times \text{VF}\;(\text{mL})}{\text{CF}\;(\text{ppm})\times \text{VF}\;(\text{mL})+\text{CR}\;(\text{ppm})\times \text{VR}\;(\text{mL})}\times 100$$

where C_F_ and C_R_ indicate the mercury concentration in the foamate and remnant, respectively, and V_F_ and V_R_ indicate the foamate and remnant volume, respectively.

## 4. Conclusions

In this study, surfactin was found to be very effective for the separation of mercury species even at low concentrations than chemical surfactants. The major advantages of using surfactin in the separation process are the higher enrichment of mercury and the higher complexation ratio. The separation of mercury ions from solutions is more effective unlike surfactant molecules adsorb in case of contaminated soils, thereby affecting the efficiency of removal. However, decrease in the pH of the metal-surfactin solution could make the reuse of surfactin possible, which renders the process viable economically on a large scale. However, further investigation of the interference and speciation of metal ions is needed. The mechanism of metal separation by anionic surfactants in foam fractionation is by complexation. Overnight incubation of the surfactin–mercury solution, increased the percentage recovery four-fold. The foam fractionation method using the biodegradable, non-toxic and cost-effective anionic biosurfactant surfactin resulted in a higher mercury recovery due to the presence of two carboxylate groups.
